# Sorafenib-Induced Autophagy Promotes Glycolysis by Upregulating the p62/HDAC6/HSP90 Axis in Hepatocellular Carcinoma Cells

**DOI:** 10.3389/fphar.2021.788667

**Published:** 2022-02-16

**Authors:** Xiaoyu Yan, Rui Tian, Jicheng Sun, Yuanxin Zhao, Buhan Liu, Jing Su, Minghua Li, Wei Sun, Xuesong Xu

**Affiliations:** ^1^ China-Japan Union Hospital, Jilin University, Changchun, China; ^2^ Key Laboratory of Pathobiology, Ministry of Education, Department of Pathophysiology, College of Basic Medical Sciences, Jilin University, Changchun, China; ^3^ Department of Molecular Biology, College of Basic Medical Sciences Jilin University, Changchun, China; ^4^ Jilin Province Zebrafish Genetic Engineering Laboratory, Jilin Province Development and Reform Commission, Jilin, China

**Keywords:** autophagy, glycolysis, p62, HDAC6, hepatocellular carcinoma, sorafenib

## Abstract

Sorafenib has attracted much attention as the first drug approved by the FDA for the treatment of advanced hepatocellular carcinoma (HCC). Because of the drug tolerance, the overall outcomes were far from satisfactory. Current studies suggest that changes in glucose metabolism induced by sorafenib are the pivotal resistant mechanism of HCC cells, but the specific regulatory mechanism remains unclear, which makes it difficult to increase drug sensitivity by targeting glycolysis. As a metabolic-recycling pathway, autophagy regulates multiple important pathways involved in cell survival and death. In this study, we found the expression of key autophagy proteins were closely related to the prognosis and progression of HCC patients. Based on *in vitro* experiments, our studies showed sorafenib induced autophagy in HCC cells. Inhibition of autophagy by chloroquine could significantly increase the sensitivity of HCC cells to sorafenib and reverse the enhancement of glycolysis. Furthermore, sorafenib-induced autophagy promoted the deacetylase activity of HDAC6 by degrading p62, which promoted the activity of PKM2 by regulating the acetylation of its critical substrate HSP90. In this study, we investigated the role of autophagy-induced HDAC6 in regulating the key glycolytic enzyme PKM2, which may be helpful to clarify the relationship between autophagy and glycolysis in a sorafenib-resistant mechanism. Targeting p62/HDAC6/HSP90 could herald a potential improvement in HCC therapy.

## Introduction

Hepatocellular carcinoma (HCC) is one of the deadliest malignant tumors. To date, no systematic treatment has been clearly proved to improve the survival rate of patients with advanced HCC. Sorafenib has attracted substantial attention as the first drug approved by the United States Food and Drug Administration for the treatment of advanced HCC (Llovet et al., 2008). It is believed that targeting the kinase activity in the Ras/Raf/MEK/ERK signaling, platelet-derived growth factor receptor (PDGFR-β) and vascular endothelial growth factor receptor (VEGFR) all contributed to its roles in tumor suppression (Fan et al., 2020). However, because of the drug tolerance, the overall outcomes were far from satisfactory. Currently, a growing body of evidence supporting energy metabolism, autophagy ATP binding box (ABC) transporters non-coding RNAs, and hypoxic microenvironment were involved in the resistant mechanism (Méndez-Blanco et al., 2019; Tang et al., 2020).

Glycolysis and oxidative phosphorylation pathways were the pivotal pathways for energy supply. Malignant tumors switched their metabolic patterns and promoted cell survival by enhancing aerobic glycolysis called the “Warburg effect”. The intermediate production during glycolysis may become precursors for the synthesis of non-essential amino acids, providing materials and energy needed for the survival and proliferation of cancer cells (Beyoğlu et al., 2013; Kee and Cheong, 2014). Recent studies indicated sorafenib may impair oxidative phosphorylation and promote the aerobic glycolysis of HCC (Li et al., 2017a; Li et al., 2017b), and sorafenib-induced adaptive alterations in glucose metabolism has occurred at multiple levels such as the abnormal activation and expression of glycolytic rate-limiting enzymes and oxidative phosphorylation mechanism defect. Furthermore, tumor heterogeneity may determine the difference in glycolytic activation with sorafenib treatment. Thus, more studies are needed to explore the mechanism of glycolysis regulation in HCC cells.

It is becoming increasingly apparent that there may exist a crosstalk between autophagy and glucose metabolism. Autophagy is the important metabolic-recycling pathway and maintains cell homeostasis by recycling misfolded and damaged proteins and organelles. It is reported that increased phosphatidylethanolamine (PE)-modified LC3/GABARAP-Ⅱ could reflect the induction of autophagic sequestration (Klionsky et al., 2021). Previous studies suggested that sorafenib promoted autophagosome formation and the higher expression of Beclin-1, Atg5, and LC3Ⅱ *in vivo* and *in vitro* (Prieto-Domínguez et al., 2016). Depending on the cell state and features, the regulatory routes were different, such as inhibition of mTORC1 complex, ER stress activation, and AMPK phosphorylation in HCC cells (Neufeld, 2010; Liu et al., 2012; Kim and Guan, 2015). Current studies found the key autophagy receptor p62/SQSTM1 has shown an abnormally high expression in a variety of tumors including HCC (Inoue et al., 2012; Choi et al., 2013; Ellis et al., 2014; Umemura et al., 2016). Furthermore, glycolytic modulators hexokinase II and LDHA were changed with p62 inhibition (Calvo-Garrido et al., 2019), which indicated p62 may be involved in the glucose metabolic shift between aerobic glycolysis and oxidative phosphorylation. Working as a signal hub, p62 interacts with several important signal molecules that respond to apoptosis, oxidative stress, and cell proliferation such as HDAC6. Increasing evidence suggested HDAC6 not only affects the fusion of autophagosomes and lysosomes but is also a histone deacetylase mainly localized in cytoplasm and regulates the acetylation of “non-histone” and mediates their stability and activity (Liu et al., 2016a).

In this study, we investigated how sorafenib-induced autophagy regulated glycolysis in HCC cells. We clarified that increased autophagy may promote HDAC6 activity by degrading HDAC6 inhibitor p62. Furthermore, activated HDAC6 inhibited HSP90 acetylation and enhanced the activity of the key glycolytic enzyme PKM2 in HCC cells. These findings increased the knowledge of a more specialized regulation mechanism between autophagy and glycolysis and provided potential targets to increase the sorafenib sensitivity of HCC cells.

## Materials and Methods

### Clinical Samples

Ninety-four pairs of liver cancer tissues and the adjacent tissues were acquired from Shanghai Outdo Biotech Co., Ltd., collected between 2007 and 2009. The tumor tissues from patients were all liver carcinoma *in situ*. The participants in this study provided informed consent for the use of their tissue samples for research. The use of tissue samples was approved by the Ethics Committee of Taizhou Hospital, Zhejiang Province. Clinical characteristics are listed in Table 1.

**TABLE1:** Clinical pathological characteristics of liver cancer cases

Characteristic	GC, N = 94	%
Age (y)		
<65	**67**	**71.3**
≥65	**18**	**19.1**
miss	**9**	**9.6**
Histological grade		
I‐III	**58**	**61.7**
III	**27**	**28.7**
miss	**9**	**9.6**
T stage		
T1‐T2	**47**	**50**
T3	**45**	**47.9**
miss	**2**	**2.1**
Follow‐ups		
Dead	**56**	**59.6**
Survival	**35**	**37.2**
miss	**3**	**3.2**

### Cell Lines and Cell Culture

SNU-449 is grade II-III/IV hepatocellular carcinoma (ATCC^®^ CRL-2234™, Manassas, VA, United States). SNU-387 is grade IV/V pleomorphic hepatocellular carcinoma (ATCC^®^ CRL-2237™, Manassas, VA, United States). SNU-387 and SNU-449 hepatocellular carcinoma cell lines were grown in DMEM (Gibco Life Technologies, Carlsbad, CA, United States) supplemented with 10% fetal bovine serum (Invitrogen, Carlsbad, CA, United States) at 37°C at 5% CO_2_ concentration.

### Reagents and Drugs

Sorafenib (Sora, HY-10201) and Tubacin (HY-13428) were purchased from Med Chem Express (Monmouth Junction, NJ, United States); Chloroquine (CQ, C6628) was purchased from Sigma Aldrich (St Louis, MO, United States).

### Cell Viability Assays

MTT assay was used to determine cell viability. Cells were seeded in 96-well plates overnight at a density of 1.2 × 104 cells/well and then treated with various reagents for the indicated times. MTT (10 µl of 10 mg/ml) reagent in phosphate-buffered saline was added to each well and incubated for 4–6 h; the formazan crystals were dissolved with 150 µl dimethylsulfoxide (DMSO). Absorbance was recorded at a wavelength of 490 nm.

### Flow Cytometry

Cells were seeded in six-well plates overnight at a density of 40 × 105 cells/well, then cells were exposed with various reagents for the indicated times. They were trypsinized and stained by Annexin V-FITC (Annexin V Apoptosis Detection Kit II; BD Biosciences, San Diego, CA, United States) to measure cellular apoptosis. The process was executed using Millipore Guava EasyCyte Flow Cytometer (France).

### Western Blot

Cells were harvested and incubated in RIPA lysis buffer for 35 min at 4°C. The concentration of proteins was measured using the Coomassie G250 assay (Beyotime Biotechnology, Shanghai, China). Equal amounts of protein lysates per well were separated by SDS-PAGE and transferred to PVDF membrane (Bio-Rad Laboratories), then they were blocked in buffer [100 mM NaCl, 10 mM Tris–HCl (pH 7.6), and 0.1% Tween 20] containing 5% non-fat dry milk for 1.5 h at room temperature. After incubation with various primary antibodies (beta-actin, p62, LC3B, HSP90, and PKM2 were obtained from Proteintech; Akt and p-4E-BP1 were obtained from Santa Cruz; ace-α-tubulin and acetyl lysine were obtained from Cell Signaling Technology) overnight at 4°C, membranes were incubated with horseradish-peroxidase-conjugated secondary antibodies (Proteintech) at room temperature for 1–2 h. Membranes were then incubated in ECL reagents (Thermo Scientific, Waltham, MA, United States) and images were captured by Syngene Bio Imaging (Synoptics, Cambridge, UK).

### Co-Immunoprecipitation

Cells were lysed in NP40 lysis buffer plus protease inhibitors. Lysates were incubated on ice for 30 min and cleared by centrifugation at 4,500 rpm for 15 min at 4°C. Lysates were incubated with antibody overnight at 4°C, followed by incubation with 25 µl protein A and G agarose (Beyotime). Beads were washed three times with 1 ml PBS and bound complexes were analyzed using immunoblotting.

### Immunofluorescence

Cells were fixed in 4% (w/v) paraformaldehyde (PFA)/PBS for 20 min and then permeabilized with 0.1% Triton X‐100 for 15 min. After blocking with bovine serum albumin for 30 min, cells were incubated with primary antibody overnight at 4°C. Cells were then incubated with FITC/Texas Red‐conjugated secondary antibodies (Proteintech) at room temperature for 1 h. The images were acquired using a Reset one inverted fluorescence microscope (Revolve; Echo Laboratories, San Diego, CA, United States).

### Plasmids and Transfection

NC, sh p62-1, and sh p62-2 (JiKai Gene, Shanghai, China) were purchased from Sangon Biotech (Shanghai, China). Cells were transfected using ViaFect™ transfection reagent according to the manufacturer’s instructions.

### Lactate Level Measurement

Cells were seeded in six-well plates. After treatment with the indicated agents, the media were collected and measured for lactate using lactate kit (Nanjing Jiancheng Bioengineering Institute, China) according to manufacturer’s protocol. Counts were normalized to protein concentration.

### Analysis of Glucose Consumption

Cells were seeded in six-well plates. After treatment with the indicated agents, the media were collected and measured for glucose using glucose kit (RsBio, Shanghai, China) according to manufacturer’s protocol. Counts were normalized to protein concentration.

### HDAC6 Activity Assay

Cells were seeded in six-well plates. After treatment with the indicated agents, using the Elisa kit assay the level of Human HDAC6 in cells (MEIMIAN, China) was determined.

### PKM2 Activity Assay

Cells were seeded in 6-well plates. After treated with the indicated agents, using the Elisa kit assay the level of Human PKM2 in cells (MEIMIAN, China) was determined.

### ATP Measurements

The ATP Bioluminescence Assay Kit (Beyotime Technology) was used to measure the level of ATP in cells. Briefly, cells were lysed with a lysis buffer, followed by centrifugation (10,000×*g*, 2 min) at 4°C. Then, the level of ATP was determined by mixing 10 µl of the supernatant with 100 µl of luciferase reagent. The emitted light was measured using a luminometer (BMG LABTECH Omage, GER). Counts were normalized to protein concentration.

### Oxygen Consumption Rates

According to manufacturer’s protocol, cells were seeded in 384-well plates overnight at a density of 8 × 103 cells/well. Then cells were exposed to various reagents. Each treatment was repeated in three wells. The determination of cell oxygen consumption rates was carried out using the fluorescent oxygen-sensitive probes Mito-Xpress (Luxcel Bioscience, Cork, Ireland), respectively.

### Extracellular Acidification Rate

According to manufacturer’s protocol, cells were seeded in 384-well plates overnight at a density of 8 × 103 cells/well, then cells were exposed to various reagents. Each treatment was repeated in three wells. Cells were then kept in a non-CO_2_ incubator at 37°C for 1 h. The determination of extracellular acidification rate was carried out using the fluorescent pH-sensitive probes Mito-Xpress and pH-Xtra (Luxcel Bioscience, Cork, Ireland), respectively.

### Statistical Analysis

Three replicates of every experiment were performed. Data are expressed as the mean ± standard (SD). Student’s *t*-test was used to assess the statistical significance of the difference between two groups. The relationship between the expression of two proteins was analyzed using Pearson correlation analysis. p < 0.05 was defined as statistically significant.

## Results

### Autophagy Activation was Rrelated to Sorafenib Sensitivity of HCC Cells

**FIGURE 1:**
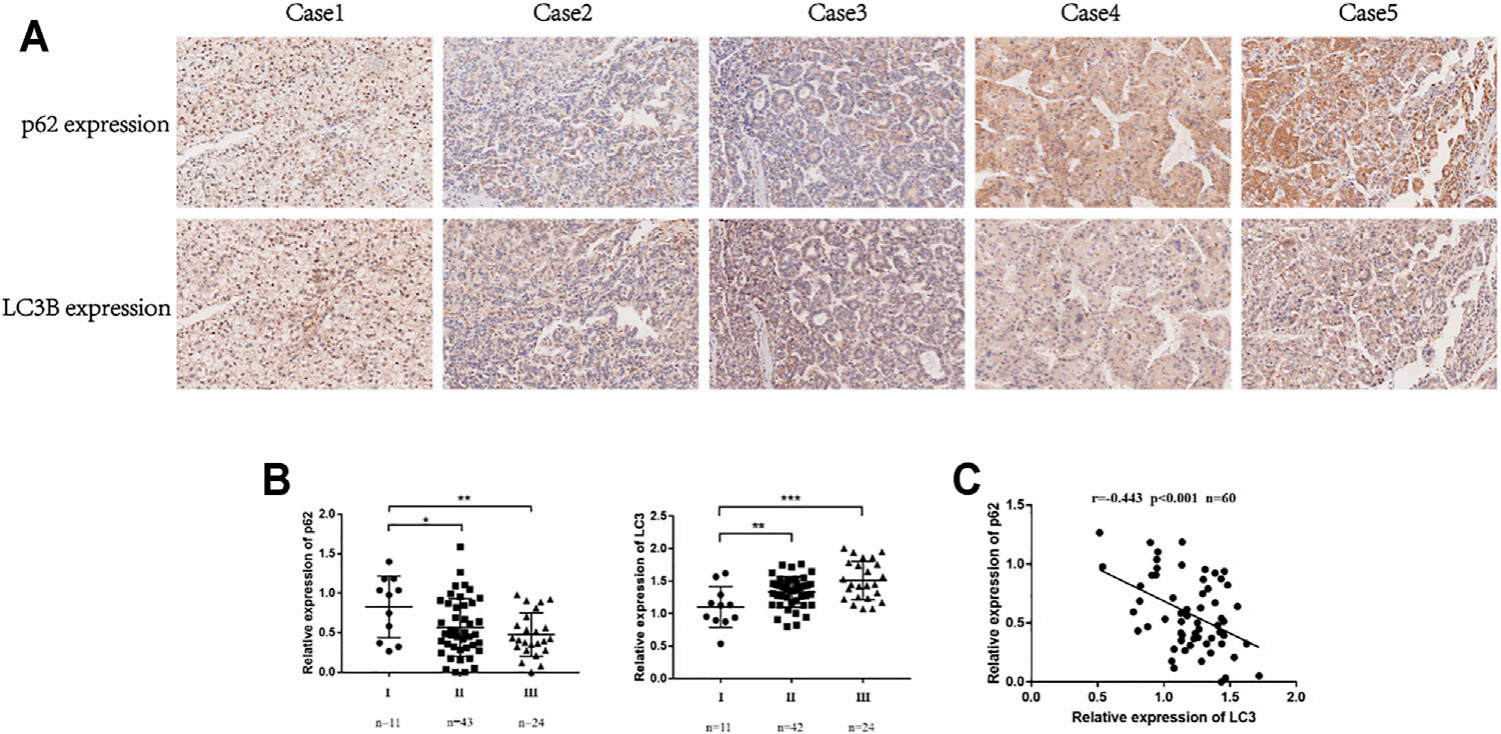
The expression of p62 and LC3 was related to the prognosis of HCC patients. **(A)**. Representative IHC for p62 and LC3B expression in tissue microarrays (100×). **(B)**. Correlation between p62 expression and TNM stages in HCC tissues. Correlation between LC3 expression and TNM stages in HCC tissues. Statistical significance was determined using a two‐tailed, unpaired Student’s *t*-test. *p *<* 0.05, **p *<* 0.01, ***p *<* 0.001. **(C)**. Pearson correlation analysis between p62 and LC3B expression in HCC tissues (*r* = −0.442, p *<* 0.001).

This study used the key autophagy receptor protein p62 and autophagosome membrane protein LC3 to determine the autophagy level of HCC cells; low expression of p62 and high expression of LC3 are indicative of a smooth autophagy flux, and accumulation of p62 and LC3 in the cell indicates the blockage of autophagosome clearance (Jiang and Mizushima, 2015). We analyzed the tumor tissues of 94 HCC patients and performed immunohistochemical staining (Figure 1A). Studies have found decreased p62 and increased LC3 expression may indicate a more malignant type (Figure 1B). The Pearson correlation analysis revealed that the expression of p62 and LC3B were negatively correlated (Figure 1C). These results suggested that autophagy level was associated with poor prognosis and progression of HCC patients.

**FIGURE 2:**
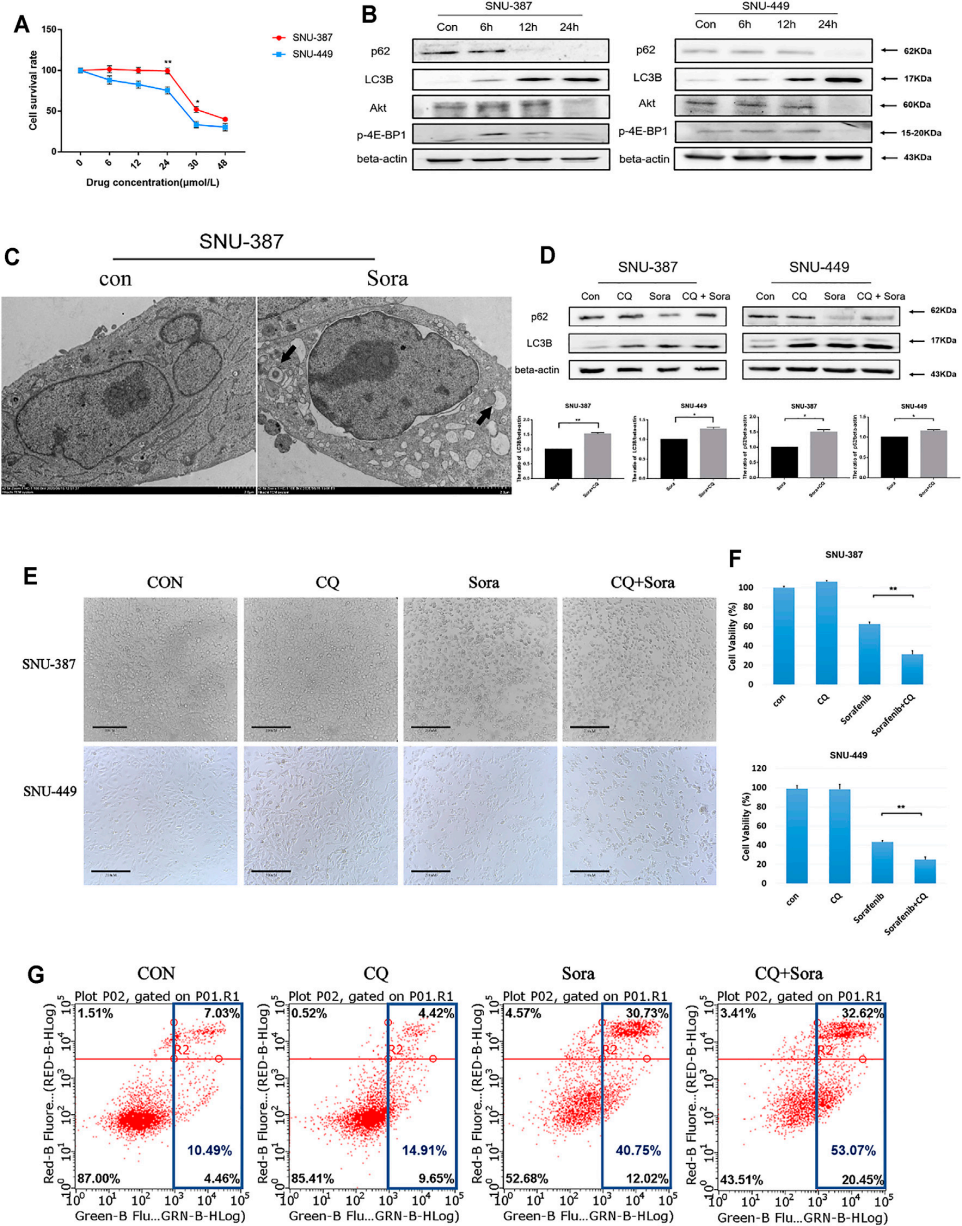
Autophagy activation was related to the decreased sorafenib sensitivity of HCC cells. **(A)**. MTT was used to detect the survival rate of SNU-387 and SNU-449 cells with sorafenib treatment. Data are presented as mean ± SD, n = 3. *p *<* 0.05, **p *<* 0.01. **(B)**. SNU-387 and SNU-449 cells were treated with sorafenib and the protein expression was analyzed by Western blotting. **(C)**. TEM was used to observe the autophagosome with sorafenib treatment in SNU-387 cells. **(D)**. SNU-387 and SNU-449 cells were treated with sorafenib for 12 h, and CQ was added in the last 4 h to block autophagy flux. p62 and LC3B were analyzed by Western blotting. Data are presented as mean ± SD, n = 3. *p *<* 0.05, **p *<* 0.01. **(E)**. SNU-387 and SNU-449 cells were treated as before and a microscope was used to observe cell morphology. **(F)**. Cell viability was detected by MTT assay. Data are presented as mean ± SD, n = 5. ****p *<* 0.0001. **(G)**. Apoptosis was detected by flow cytometry.

To verify the relationship between autophagy and sorafenib sensitivity, two HCC cell lines with different malignant degrees were used. The results showed that SNU-387 cells were less sensitive to sorafenib (Figure 2A). Western blotting found p62 protein was suppressed, whereas LC3B protein expression was increased, suggesting sorafenib may induce autophagy in HCC cells. Simultaneously, the expression of p-4E-BP1 downstream of Akt/mTOR pathway decreased gradually with sorafenib treatment. Current studies found the activation of mTOR could inhibit autophagy by phosphorylating a variety of autophagy-related proteins such as ULK1, ATG13, AMBRA1, and ATG14L, which suggested sorafenib-induced mTOR inhibition may be responsible for autophagy enhancement (Figure 2B) (Kim and Guan, 2015). Furthermore, autophagosome was obviously shown under electron microscopy (Figure 2C). Subsequently, autophagy inhibition experiments found blocking the autophagy flux caused increasing LC3B accumulation in HCC cells, which suggested higher autophagy levels in SNU-387 cells (Figure 2D). In addition, inhibition autophagy significantly increased the level of apoptosis and induced cell death (Figures 2E,F). These studies may indicate that sorafenib could induce stronger autophagy in malignant HCC cells and was related to the decreased sorafenib sensitivity.

### Sorafenib-Induced Autophagy was Responsible for Increased Glycolysis in HCC Cells

**FIGURE 3:**
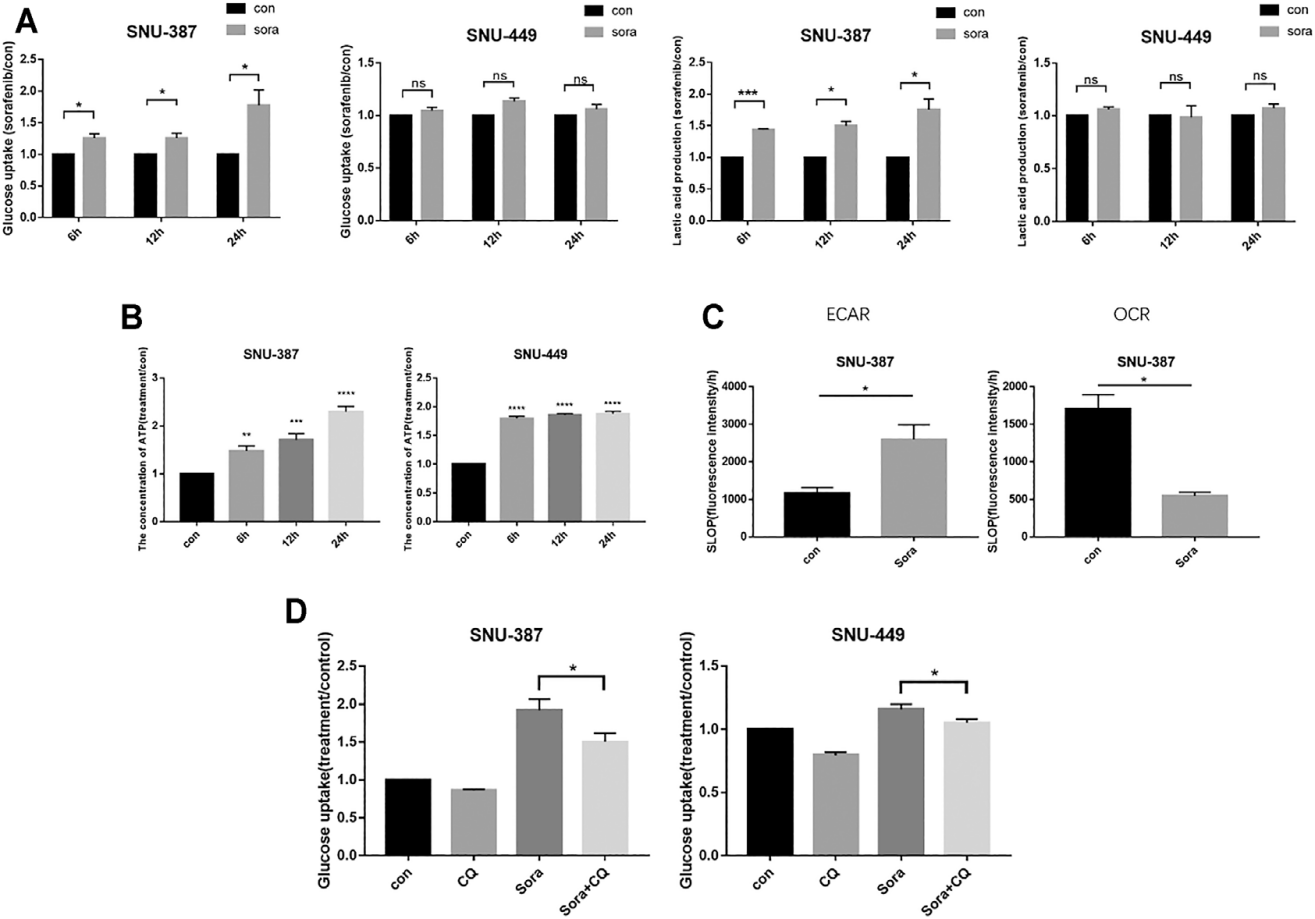
Sorafenib-induced autophagy was responsible for increased glycolysis in HCC cells. **(A)**. SNU-387 and SNU-449 cells were treated with Sorafenib and the glucose consumption and lactic acid production were detected. Data are presented as mean ± SD, n = 3. *p *<* 0.05. **(B)**. Cells were treated as before and the production of ATP was detected. Data are presented as mean ± SD, n = 3. **p < 0.01, ***p < 0.001, ****p < 0.0001. **(C)**. The results of ECAR and OCR were quantified to analyze glucose and oxygen consumption with Sorafenib treatment. Data are presented as mean ± SD, n = 3. *p < 0.05. **(D)**. SNU-387 and SNU-449 cells were treated with autophagy inhibitor CQ, and glucose consumption was analyzed. Data are presented as mean ± SD, n = 3. *p *<* 0.05 **p *<* 0.01.

We further examined glycolysis with sorafenib treatment in HCC cells. The results suggested that the glucose consumption and lactate production of SNU-387 cells significantly increased with sorafenib treatment, while ATP production was also drug-gradient dependent (Figures 3A,B). Importantly, extracellular acid and oxygen consumption results indicated increasing glycolysis level and decreasing oxygen consumption rate in SNU-387 cells (Figure 3C). Autophagy inhibition downregulated the glucose consumption of SNU-387 (Figure 3D). These results indicated sorafenib-induced autophagy may contribute to increased glycolysis in HCC cells.

### Sorafenib-Induced Autophagy Could Restore the Deacetylase Activity of HDAC6 Through Promoting p62 Degradation

**FIGURE 4:**
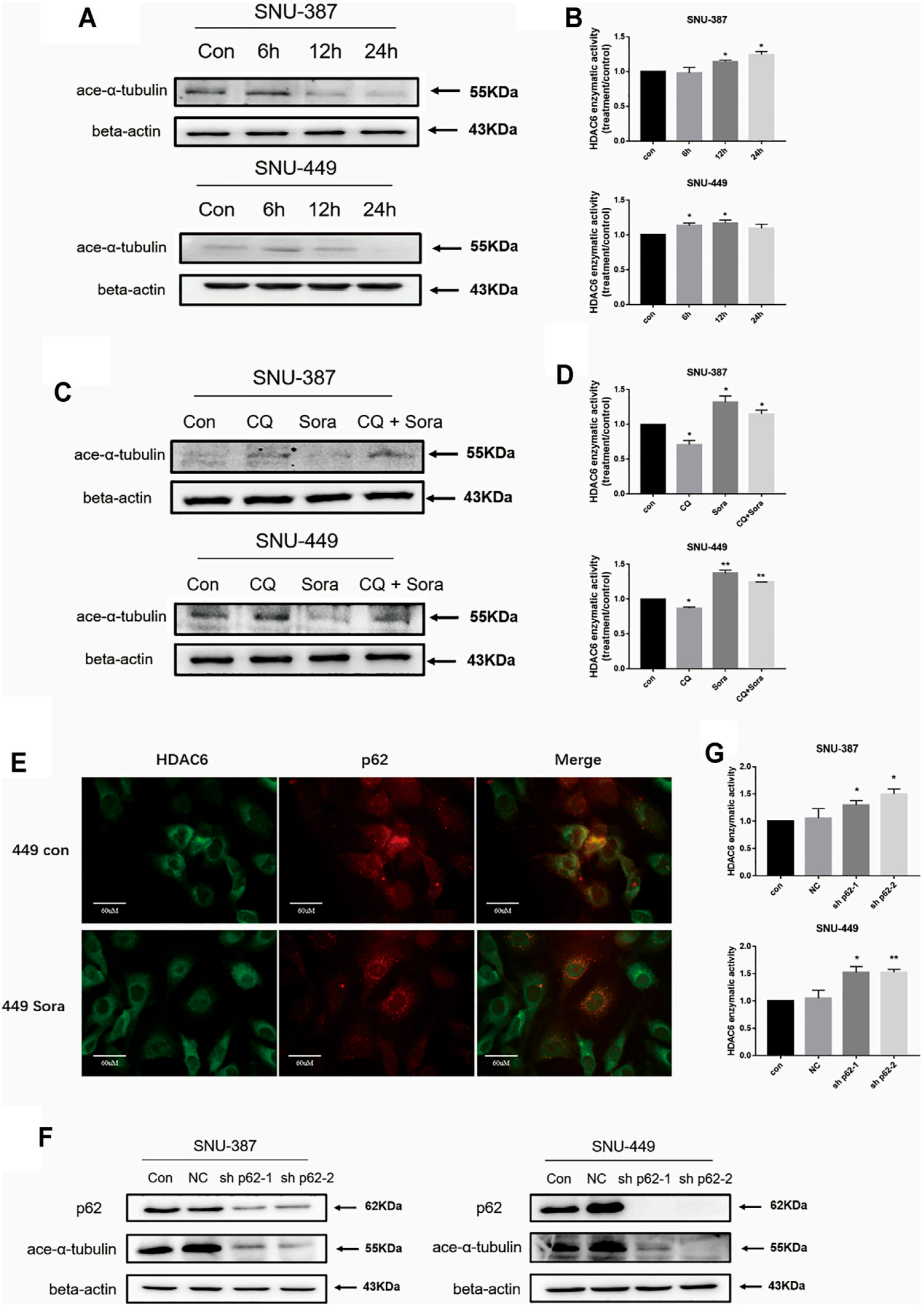
Sorafenib-induced autophagy restored the deacetylase activity of HDAC6 through promoting p62 degradation. **(A)** SNU-387 and SNU-449 cells were treated as before and the expression of ace-α-tubulin was analyzed by western blotting. **(B)** The enzyme activity of HDAC6 was detected by ELISA kit. Data are presented as mean ± SD, n = 3. *p < 0.05. **(C)** The expression of ace-α-tubulin in SNU-387 and SNU-449 cells lines were analyzed by western blotting with Sorafenib and CQ treatment. **(D)** The enzyme activity of HDAC6 was detected by Elisa assay. Data are presented as mean ± SD, n = 3. *p < 0.05, **p < 0.01. **(E)** Localization of p62 and HDAC6 was observed with microscopy (Scale bar, 60 μm). **(F)** The expression of ace-α-tubulin was analyzed by western blotting with sh-p62 transfection. **(G)** The enzyme activity of HDAC6 was detected with Elisa assay. Data are presented as mean ± SD, n = 3. *p < 0.05, **p < 0.01.

Previous studies suggested HDAC6 is one of the partner molecules of p62, and its activity is closely regulated by p62 (Calvo-Garrido et al., 2019). In order to measure HDAC6 activity, we analyzed the acetylation level of its typical substrate α-tubulin. The results found α-tubulin acetylation was significantly reduced in SNU-387 cells. Consistent with its results, activity of HDAC6 was significantly enhanced with sorafenib treatment. In addition, autophagy inhibition could significantly reduce HDAC6 activity (Figures 4C,D). Immunofluorescence showed the co-localization of p62 and HDAC6 was suppressed when treated with sorafenib (Figure 4E). In order to verify the role of p62 in HDAC6 regulation, we found p62 inhibition could obviously suppress HDAC6 activity (Figures 4F,G). These results indicated that sorafenib-induced autophagy may regulate HDAC6 through p62 inhibition in HCC cells.

### HDAC6 Enhanced the HSP90 and PKM2 Interaction With Sorafenib Treatment in HCC Cells

**FIGURE 5:**
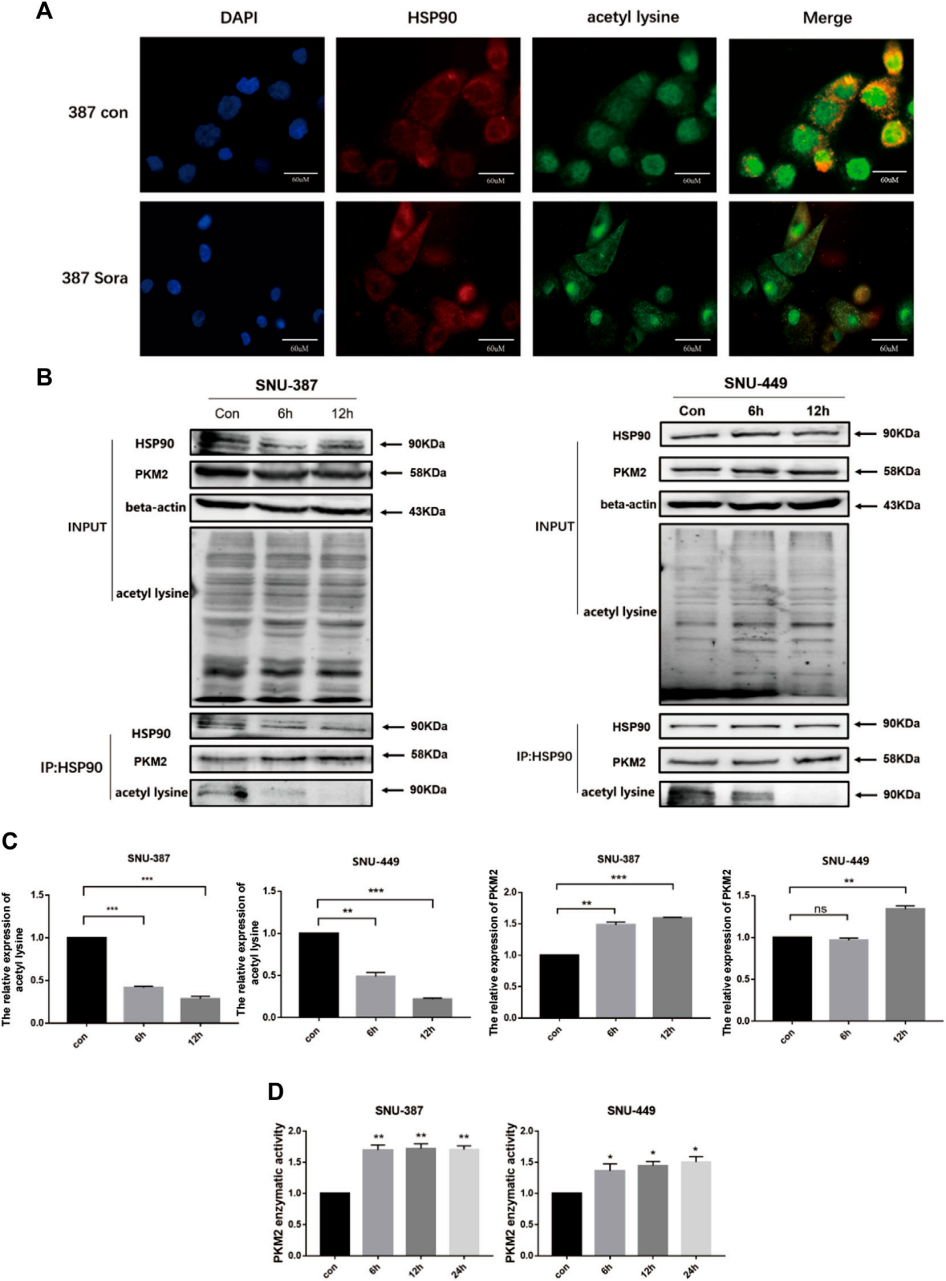
HDAC6 enhanced the activity of PKM2 by regulating HSP90 acetylation. **(A)** HSP90 acetylation was detected by immunofluorescence (Scale bar, 60 μm). **(B-C)** Immunoprecipitation was performed with the anti-HSP90 antibody followed by western blotting using anti-PKM2, anti-acetyl lysine. Quantification of PKM2 and acetyl lysine were shown. Each bar represents the mean ± SD **p < 0.01, ***p < 0.001. **(D)** The activity of PKM2 was detected after treatment with sorafenib treatment in SNU-387 and SNU-449 cells. Data are presented as mean ± SD, n = 3. **p < 0.01.

Recent studies found HSP90 was involved in protein folding and maturation including critical enzyme of glycolysis such as PKM2. The excessive acetylation of HSP90 may destroy its role in maintaining client protein maturation and stability (Xu et al., 2017). Immunoprecipitation and immunofluorescence results showed sorafenib reduced the acetylation of HSP90 in HCC cells. Furthermore, the combination between HSP90 and PKM2 was enhanced (Figures 5A–D). In addition, with the prolongation of sorafenib treatment, the enzymatic activity of PKM2 was significantly increased (Figure 5E).

### p62/HDAC6/HSP90 Axis Contributed to Crosstalk Between Sorafenib-Induced Autophagy and Glycolysis in HCC Cells

**FIGURE 6:**
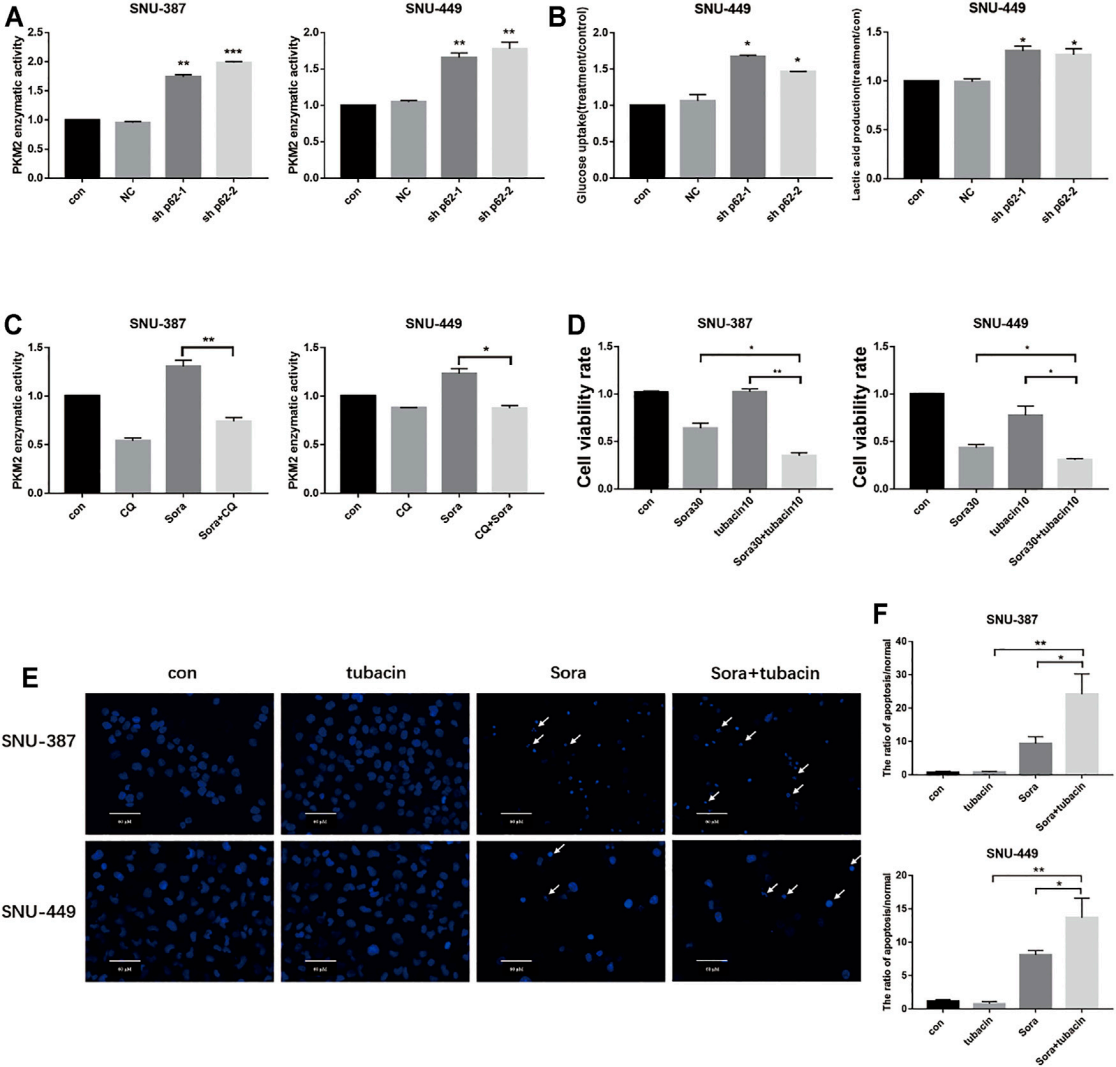
Sorafenib-induced autophagy promoted PKM2 through the p62/HDAC6/PKM2 axis. **(A)**. SNU-387 and SNU-449 cells were transfected with sh-p62. PKM2 enzyme activity was analyzed by Elisa assy. Data are presented as mean ± SD, n = 3. **p *<* 0.01, ***p *<* 0.001*.* **(B)**. Glucose consumption and lactic acid production were detected in HCC cells. Data are presented as mean ± SD, n = 3. *p *<* 0.05. **(C)**. The enzyme activity of PKM2 was examined with Sorafenib and CQ treatment. Data are presented as mean ± SD, n = 3. *p *<* 0.05. **(D)**. SNU-387 and SNU-449 cells were treated with Sorafenib and an HDAC6 inhibitor tubacin. MTT was used to detect the cell viability. Data are presented as mean ± SD, n = 3. *p < 0.05, **p < 0.01. **(E,F)**. SNU-387 and SNU-449 cells were stained with Hoechst 33342. Cell morphology was observed using microscopy. Arrows indicate apoptotic cells. Scale bar, 60 μm. Data are presented as mean ± SD, n = 3. *p < 0.05, **p < 0.01.

**FIGURE 7:**
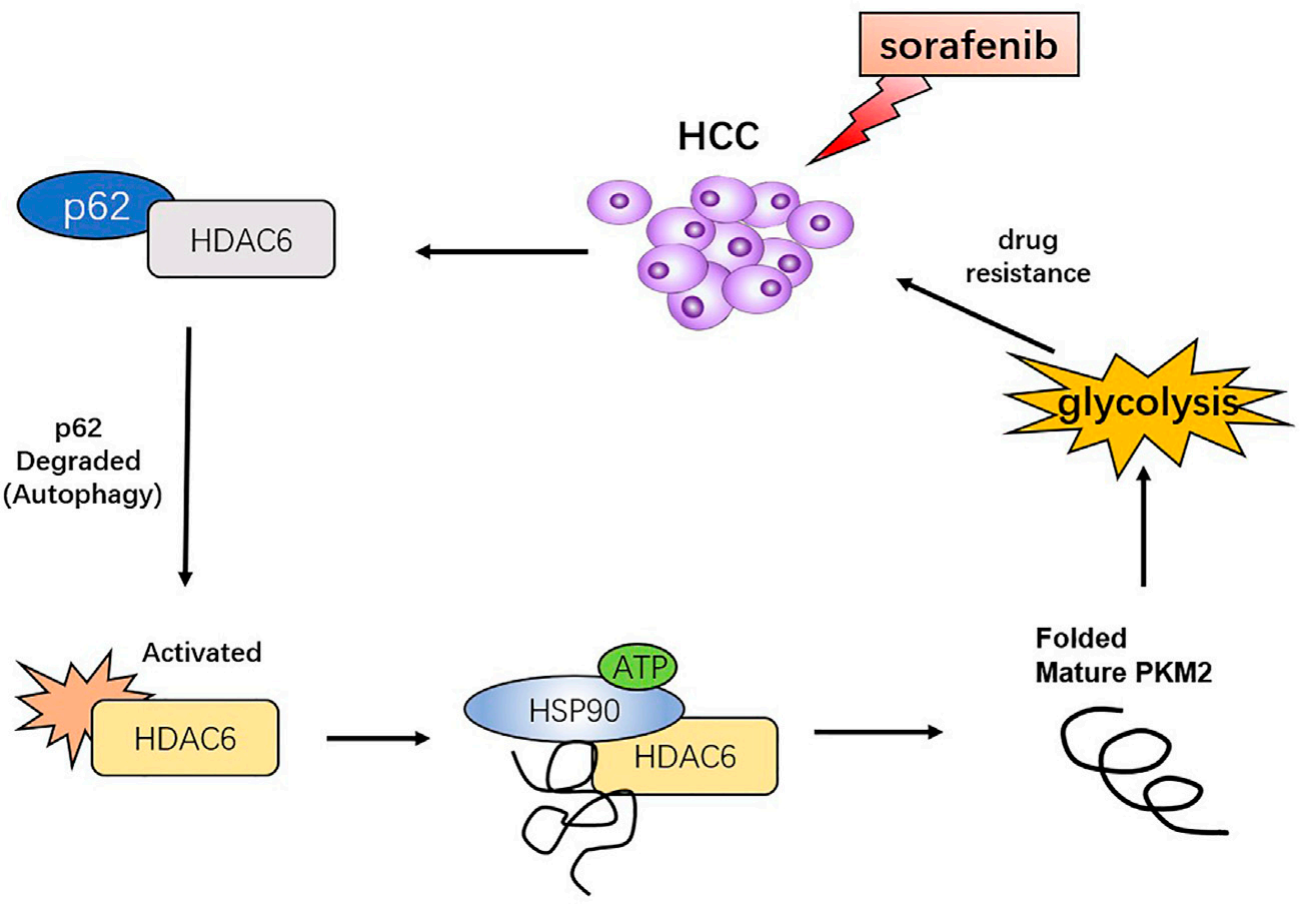
Proposed model that sorafenib-induced autophagy promotes the activity of glycolysis enzyme PKM2 through p62/HDAC6/HSP90 axis in HCC cells.

To further elucidate the role of autophagy in PKM2 regulation, we inhibited p62 in HCC cells and found that not only lactic acid production but also PKM2 enzyme activity was significantly increased (Figures 6A,B). In addition, autophagy inhibition could suppress PKM2, which indicated that sorafenib-induced autophagy may promote glycolysis through upregulating PKM2 activity (Figure 6C). Tubacin is a reported highly selective HDAC6 inhibitor which belongs to aliphatic-chain hydroxamate family (Yan et al., 2021). We found suppressed HDAC6 activity with Tubacin could increase the sensitivity of sorafenib in HCC cells (Figures 6D–F). These findings suggested p62/HDAC6/HSP90 axis was responsible for crosstalk between sorafenib-induced autophagy and glycolysis in HCC cells (Figure 7).

## Discussion

In this study, we clarified the role of sorafenib-induced autophagy in the activation of glycolytic signals in HCC cells by studying the mechanism of autophagy protein p62/HDAC6/HSP90 in regulating the key glycolytic enzyme PKM2. Our research provides a new theoretical basis for targeting p62/HDAC6/HSP90 in order to enhance the sorafenib sensitivity of HCC cells.

The multi-kinase inhibitor sorafenib is approved for the treatment of advanced HCC. However, the poor response of patients has become an obstacle in HCC therapy. Currently, sorafenib-induced autophagy has been proved to be the critical resistant mechanism, which not only promotes the catabolism of HCC cells but also plays key roles in coordinating energy metabolism. (Shi et al., 2011; Shimizu et al., 2012; Yuan et al., 2014; Zhai et al., 2014; Ling et al., 2017). Increasing evidence showed that metabolic flexibility may be responsible for tumors to go against chemotherapy induced stress. Gao et al. performed whole transcriptome analysis in sorafenib-resistant HCC cells, which suggested HIF1α-related signaling may be activated as a major pathway (Gao et al., 2021). Another study found glycolysis was enhanced in response to sorafenib treatment, and high levels of glycolysis indicated poor prognosis in chemotherapy (Feng et al., 2020). In addition, miRNA-mediated inhibition of rate-limiting enzymes in glycolytic pathway has come into focus recently (Fu et al., 2020). Our study found sorafenib treatment significantly increased the glucose uptakes and lactic acid production in SNU-387 cells. Glycolysis was significantly decreased while autophagy inhibition, suggesting that sorafenib-induced autophagy may participate in glucose metabolism reprogramming of HCC cells, which is closely related to the drug-resistant mechanism.

A previous study found that the autophagy receptor protein p62 played a key role in cisplatin resistance of ovarian cancer by regulating the autophagy flux (Yan et al., 2019). We analyzed the tumor tissues from HCC patients and found that the expression of p62 and LC3B was closely related to the pathological stage of HCC, which indicated autophagy may contribute to HCC procession. It is becoming increasingly apparent that autophagy functions as a double-edged sword. It may suppress tumorigenesis in the early stage through initialing damaged proteins or organelle clearance and turn into an accomplice through controlling energy metabolism while cancer is established (Wong et al., 2021). In this study, we found inhibiting sorafenib-induced autophagy promoted cell death, which indicated autophagy may protect HCC cells that escape from drug damage. These are intriguing lines of research that p62 promotes the formation of protein aggregates and participates in the regulation of autophagy (Liu et al., 2016a), and it also acts as a molecular hub to interact with a variety of proteins involved in tumor survival. Recent studies have found that the functional domain of p62 may directly bind to HDAC6 (Liu et al., 2016a). Our experiments showed that sorafenib promoted the degradation of p62 and upregulated the enzymatic activity of HDAC6. We thought sorafenib-induced HDAC6 activity may play a protective role against drug damaging as HDAC6 inhibition could increase the sensitivity of sorafenib in HCC cells. Immunofluorescence revealed that sorafenib treatment significantly inhibited the co-localization between p62 and HDAC6. Furthermore, suppressed p62 expression increased the deacetylase activity of HDAC6 in HCC cells, suggesting that sorafenib-induced autophagy may upregulate HDAC6 activity by degrading p62.

It is shown that the ATPase activity of HSP90 is essential for maintaining the stability and function of onco-proteins, and its acetylation was involved in controlling the affinity with the clients (Kamal et al., 2003; Yang et al., 2015). The data from Protein Atlas (www.proteinatlas.org) indicated that high expression of PKM2 was related to poor prognosis in liver cancer. It is becoming increasingly apparent that PKM2, the participant in the glucose reprogramming and drug resistance of tumor cells, may be the potential substrate of HSP90 (Taipale et al., 2010; Zuo et al., 2012; Asthana et al., 2013; Liu et al., 2016b). Christofk et al. showed that decreased PKM2 can obviously inhibit the glycolysis level (Christofk et al., 2008). In addition, Bluemlein et al. also demonstrated that dynamic changes of PKM2 activity could affected the Warburg effect of tumor cells (Bluemlein et al., 2011). Our studies found that sorafenib significantly reduced the acetylation level of HSP90 in HCC cells. Previous studies demonstrated HSP90 could bind with PKM2 and regulate the phosphorylation and protein stability of PKM2 (Xu et al., 2017). Our results showed the up-regulation of PKM2 activity induced by sorafenib treatment may blame to the interacting enhancement of HSP90 and PKM2, which indicated deacetylation of HSP90 may play a pivotal role in up-regulating the activity of PKM2.

In summary, our research determined that the p62-HDAC6-HSP90 pathway was involved in the crosstalk between autophagy and glycolysis in HCC cells. Interestingly, this mechanism may favor highly malignant HCC cells over tumor cells from patients in the early stage. Our studies indicated HDAC6 inhibitor may become potentially adjuvant to enhance the sensitivity of sorafenib in HCC patients with higher levels of autophagy and glycolysis. Interestingly, it suggested that HDAC inhibitor could also improve the anti-tumor effects of other approved multi-kinase inhibitors such as lenvatinib, which indicated that the crosstalk between autophagy and glycolysis may also play pivotal roles in a lenvatinib-resistant mechanism (Roberts et al., 2020). In the future study, we intend to evaluate the feasibility of targeting p62/HDAC6/HSP90 to increase the sensitivity of sorafenib based on *in vivo* experiments to maximize patient outcomes in this new rapidly shifting landscape.

## Data Availability

The original contributions presented in the study are included in the article/supplementary material. Further inquiries can be directed to the corresponding author.
